# Comparison of Opioid-Based Versus Opioid-Sparing Anesthesia in Patients Undergoing Glioma Surgery

**DOI:** 10.7759/cureus.54153

**Published:** 2024-02-13

**Authors:** Anupama A S, Ashutosh Kaushal, Vaishali Waindeskar, Saurabh Saigal, Anuj Jain, Harish Kumar, Pranita Mandal, Sandeep Kumar, Sweta Kumari, Anjan K Sahoo

**Affiliations:** 1 Anaesthesiology, All India Institute of Medical Sciences, Bhopal, Bhopal, IND; 2 Microbiology, All India Institute of Medical Sciences, Bhopal, Bhopal, IND; 3 Otolaryngology - Head and Neck Surgery, All India Institute of Medical Sciences, Bhopal, Bhopal, IND

**Keywords:** mean arterial pressure, heart rate, dexmedetomidine, fentanyl, opioid free anesthesia

## Abstract

Background

In the neurosurgical population, opioids may cause respiratory depression, leading to hypercapnia, increased cerebral blood flow (CBF), and ultimately increased intracranial pressure (ICP), which can mask early signs of intracranial complications and delayed emergence. This study was designed to compare perioperative hemodynamic stability, analgesia, and recovery parameters in opioid-based (fentanyl) general anesthesia versus opioid-sparing (dexmedetomidine) general anesthesia in patients undergoing glioma surgeries.

Methodology

This prospective observational comparative study compared 52 patients in two groups. Twenty-six (50%) patients in group F received Inj. fentanyl IV (intravenous; bolus 2 mcg/kg 10 minutes before induction and then infusion 1 mcg/kg/hour till 30 minutes before skin closure), whereas 26 (50%) patients in group D received Inj. dexmedetomidine IV (0.5 mcg/kg infusion 10 minutes before induction and then maintenance with a 0.5 mcg/kg/hour infusion till 30 minutes before skin closure). Perioperative heart rate (HR), mean arterial pressure (MAP), Numerical Rating Scale for Pain (NRS) assessment and postoperative emergence time, modified Aldrete score, patient satisfaction, and surgeon satisfaction score were estimated and compared in both groups.

Results

The mean HR was less in group D compared to group F at following time points - 10 minutes after infusion (*P *= 0.006), laryngoscopy and intubation (*P *= 0.003), pinning of the skull (*P *< 0.001), one hour after dura opening (*P *= 0.007), two hours after dura opening (*P* = 0.006), five minutes after extubation (*P *< 0.001), and 30 minutes after extubation (*P *= 0.011). MAP was lower in group D compared to group F at the following time intervals: 10 minutes after infusion (*P *= 0.008), five minutes after extubation (*P *= 0.007), 30 minutes after extubation (*P* < 0.001), and one hour after extubation (*P *= 0.023). A significant decrease in emergence time in group D compared to group F (*P *< 0.001) was noted. NRS was lower in group D at eight hours (*P *= 0.005) and 12 hours (*P *< 0.001) post-extubation.

Conclusions

Dexmedetomidine can be used as an alternative to fentanyl in terms of perioperative hemodynamic stability, perioperative analgesia, smooth early recovery from anesthesia, patient satisfaction, and surgeon satisfaction.

## Introduction

The goal of neuroanesthesia for glioma surgery is to provide stable systemic and cerebral hemodynamics, good operating conditions, adequate analgesia, and rapid recovery to enable quick and early neurological testing [1-4].

Opioids are the most widely used analgesic agent for treating moderate-to-severe perioperative pain due to craniotomy. In the neurosurgical population, opioids may cause increased postoperative nausea and vomiting (PONV), respiratory depression, prolonged sedation leading to hypercapnia, increased cerebral blood flow, and ultimately increased intracranial pressure, which can mask early signs of intracranial complications and delayed emergence [5]. Due to these potential adverse effects associated with opioids, there is emerging interest in exploring opioid-sparing anesthesia and perioperative analgesia during neurosurgery [6].

Dexmedetomidine is an alpha-2 agonist, gaining popularity in neuroanesthesia as an adjuvant for its sympatholytic, sedative, and hemodynamic stabilizing properties without causing significant respiratory depression and a decrease in the need for anesthesia [7-9]. In one of the recent meta-analyses, dexmedetomidine was proven to be better at preventing tachycardia following endotracheal intubation than fentanyl [10].

There is a literature gap regarding the comparison of fentanyl with dexmedetomidine as an adjunct to general anesthesia in terms of hemodynamic stability and analgesia during intubation and craniotomy for glioma surgery.

The primary objective of this study was to estimate and compare perioperative hemodynamic stability (heart rate [HR] and mean arterial pressure [MAP]) in opioid-based (fentanyl) general anesthesia versus opioid-sparing (dexmedetomidine) as the primary systemic analgesic in patients undergoing glioma surgeries. The secondary objectives were to estimate and compare the doses of additional intraoperative fentanyl bolus, emergence time, modified Aldrete score, numerical rating scale (NRS) for pain assessment, patient satisfaction, and surgeon satisfaction between the groups.

## Materials and methods

This prospective nonrandomized comparative study was undertaken in 52 patients undergoing glioma surgeries in a tertiary care center after obtaining approval from the institutional ethical committee (IHEC-PGR/2021/DM/Mch/Jan 08, 2021, and August 28, 2021) and the Clinical Trial Registry - India (CTRI/2022/01/039670). Written informed consent was obtained from patients or responsible kin meeting the inclusion criteria.

Patients aged 18 to 65 years, classified under the American Society of Anesthesiologists (ASA) physical status I and II, and scheduled for elective glioma surgery were eligible for inclusion. Exclusion criteria encompassed nonconsenting patients, those with psychiatric conditions, and individuals experiencing hepatic, renal, or cardiac dysfunction. Additionally, patients with an HR below 50 beats per minute, cardiac arrhythmias, known allergies to dexmedetomidine, and those not extubated in the operating room were excluded from the study.

All patients were examined the evening before surgery and were familiarized with the numerical rating scale (NRS) for pain assessment, and baseline NRS was noted. Standard anesthesia technique was followed in both groups. On arrival in the operation theater, the patient’s identity and nil-by-mouth status consent were confirmed. Standard ASA monitoring (electrocardiogram [ECG], noninvasive blood pressure (NIBP), peripheral capillary oxygen saturation (SPO_2_), and temperature probe) was attached, and baseline preoperative values of these parameters were recorded. Under strict aseptic precautions, a left radial artery was cannulated under local anesthesia for invasive arterial blood pressure monitoring. Patients in group F received fentanyl intravenous (IV, 2 mcg/kg [total body weight] infusion for 10 minutes before induction and then 1 mcg/kg/hour infusion till 30 minutes before skin closure), whereas patients in group D received dexmedetomidine IV (0.5 mcg/kg infusion 10 minutes before induction and then 0.5 mcg/kg/hour infusion till 30 minutes before skin closure) [11].

In both groups, anesthesia was induced with IV propofol titrated with the loss of verbal command. After achieving neuromuscular blockade with IV vecuronium 0.10 mg/kg, direct laryngoscopy and intubation of the trachea with an appropriate-size endotracheal tube were performed. A bilateral scalp block using Inj. Bupivacaine 0.5% was conducted in both groups following intubation. Additionally, Inj. paracetamol 1 g IV was administered to both groups after intubation.

Anesthesia was sustained using a mixture of oxygen and nitrous oxide in a ratio of 40%:60%, supplemented with isoflurane to attain a minimum alveolar concentration of 0.5 to 1. Neuromuscular block was maintained with continuous infusion of IV vecuronium at 1 mcg/kg/minute. Intermittent positive pressure ventilation with a tidal volume of 6-8 mL/kg body weight was instituted to maintain end-tidal carbon dioxide between 30 and 35 mmHg. The depth of anesthesia was adjusted to keep the Bispectral index value between 40 and 60. The respective drug infusion was discontinued at around 30 minutes before the end of surgery. After the skin closure, Isoflurane was discontinued, and the patients were ventilated with 100% oxygen. IV administration of Inj. Ondansetron, 4 mg, as a prophylactic antiemetic agent, took place in all patients 30 minutes before extubation. Neuromuscular blockade was reversed using IV Neostigmine 50 mcg/kg and Glycopyrrolate 10 mcg/kg. The patients were extubated when respiration was regular and adequate and patients could open their eyes spontaneously or obey simple commands. Emergence time was defined as the duration between the discontinuation of Isoflurane and the occurrence of either eye-opening or the patient spontaneously obeying verbal commands.

Intraoperatively, bradycardia (HR < 20% from baseline), tachycardia (HR > 20% from baseline), hypertension (MAP > 20% from baseline), and hypotension (MAP < 20% from baseline) were recorded and treated. IV atropine 0.6 mg was administered to treat bradycardia, whereas tachycardia was treated with an additional bolus of Inj. fentanyl 50 mcg IV and an IV bolus dose of esmolol (100 mcg/kg). Hypotension was treated with a bolus dose of Inj. mephentermine 6 mg intravenously.

The hemodynamic variables, HR, and MAP were recorded at baseline, 10 minutes after bolus infusion dose, during laryngoscopy and intubation, at pinning of the skull, skin incision, bone flap removal, dural opening, one-hour regular intervals till skin closure, at extubation, 5 minutes after extubation, and then every 30 minutes interval till four hours. Other parameters recorded were emergence time and NRS.

Surgeon satisfaction scores, rated on a scale from 1 to 3 (1 for completely dissatisfied, 2 for partial satisfaction, and 3 for completely satisfied), and patient satisfaction scores, assessed using a Likert scale (ranging from 1 to 5 where 5 indicates very much satisfied, 4 indicates somewhat satisfied, 3 denotes undecided, 2 signifies not really satisfied, and 1 represents not at all satisfied), were evaluated in the postoperative period.

Time from extubation to the first request of analgesia was recorded. Postoperative analgesia was managed with Inj. paracetamol 1 g IV BD and additional Inj. paracetamol 1 g IV as rescue analgesia was administered when the NRS score was found to be 4 or more.

Since no appropriate previous data for sample size calculation was available, the sample size for the study was based on an assumed effect size of 0.8 (large effect size based on Cohen's convention).

The sample size required in each arm of the study was calculated according to the formula given by Snedecor and Cochran:

Sample size (*N*) = 1 + 2(*Zα* + *Z*1-*β*)2/*d*2

where *d* (effect size) = 0.8, type I error (*α*) = 5%, *Zα* (value of standard normal distribution for *α* = 5%) = 1.96, type II error (*β*) = 20%, power (1 - *β*) = 80%, and *Z*1-*β* = 0.842. Based on the aforementioned formula, using the mentioned values, the sample size required is:

Sample size (*N*) = 1 + 2(1.96 + 0.842)2/0.82 = 25.5 ≈ 26.

Thus, assuming 80% power and 95% confidence interval, the minimum calculated sample size for each arm was 26 (total = 52).

Statistical package for the social sciences (SPSS) v23 (IBM Corp., Armonk, NY) was used for data analysis. Descriptive statistics was elaborated as means/standard deviations and medians/interquartile ranges (IQRs) for continuous variables and frequencies and percentages for categorical variables. Group comparisons for continuously distributed data were made using the independent sample's t-test when comparing two groups and one-way analysis of variance (ANOVA) when comparing more than two groups. If data were found to be nonnormally distributed, appropriate nonparametric tests in the Wilcoxon/Kruskal Wallis test were used for these comparisons. The chi-square test was used for group comparisons of categorical data. Linear correlation between two continuous variables was explored using Pearson’s correlation (if data were normally distributed) and Spearman’s correlation (for nonnormally distributed data). Statistical significance was kept at *P* < 0.05.

## Results

A total of 52 patients (26, 50%, in each group) were included in this study. The two groups were comparable concerning age, sex, American Society of Anaesthesiologists physical status, surgery duration, and anesthesia duration, as shown in Table 1.

**Table 1:** Patient characteristics. *P *< 0.05 is considered significant. SD, standard deviation; *n*, number; ASA-PS, American Society of Anaesthesiologists physical status

Variables		Group D (*n *= 26)	Group F (*n *= 26)	*P*-value
Age (years), mean ± SD		39.15 ± 12.01	39.42 ± 11.97	0.936
Gender, *n* (%)	Male	16 (61.5%)	14 (53.9%)	0.575
	Female	10 (38.5%)	12 (26.1%)	
ASA-PS, *n* (%)	I	11 (42.3%)	16 (61.5%)	0.5
	II	15 (57.7%)	10 (38.5%)	
Duration of surgery (hours), mean ± SD		2.70 ± 0.493	2.77 ± 0.558	0.612
Duration of anesthesia (hours), mean ± SD		3.33 ± 0.605	3.26 ± 0.580	0.659

Statistically significant HR reduction was seen in group D compared to group F at the following time points: 10 minutes after bolus infusion, laryngoscopy and intubation, skull pinning, one hour and two hours after dura opening, and 5 and 30 minutes after extubation, as shown in Table 2.

**Table 2:** Comparison of heart rate (beats/minute) between the two groups. *P *< 0.05 is considered significant. SD, standard deviation; *n*, number

Heart rate (beats/minute)	Group D (*n *= 26)	Group F (*n *= 26)	*P*-value
	Mean ± SD	Mean ± SD	
Baseline	75.23 ± 3.02	75.65 ± 3.19	0.625
10 minutes after bolus infusion	65.5 ± 7.93	72.69 ± 10.14	0.006
During laryngoscopy and intubation	85.76 ± 3.86	90.31 ± 6.49	0.003
Pinning of the skull	74.03 ± 6.97	81.03 ± 5.14	<0.001
Skin incision	77.23 ± 9.11	70.50 ± 16.44	0.074
Bone flap removal	73.23 ± 8.42	71.57 ± 10.75	0.539
Dura opening	68.96 ± 6.76	70.92 ± 10.36	0.423
One hour after dura opening	66.23 ± 6.36	73.34 ± 11.09	0.007
Two hours after dura opening	67.64 ± 7.79	76.17 ± 11.57	0.006
Dura closure	73.15 ± 8.41	78.65 ± 13.06	0.077
At extubation	98.11 ± 6.44	97.15 ± 10.96	0.701
5 minutes after extubation	74.65 ± 4.52	84.11 ± 10.26	<0.001
30 minutes after extubation	75.88 ± 3.85	80.0 ± 6.92	0.011
One hour after extubation	77.0 1 ± 5.17	77.11 ± 9.29	0.956
Two hours after extubation	73.29 ± 5.41	73.96 ± 7.92	0.714

However, the HR was within 20% of the baseline. HR was comparable at other time points. Statistically significant MAP reduction was seen in group D compared to group F at the following time points: 10 minutes after bolus infusion, five minutes, 30 minutes, and one hour after extubation, as shown in Table 3.

**Table 3:** Comparison of MAP (mmHg) between group D and group F. *P *< 0.05 is considered significant. SD, standard deviation; *n*, number; MAP, mean arterial pressure

MAP (mmHg)	Group D (*n *= 26)	Group F (*n *= 26)	*P*-value
	Mean ± SD	Mean ± SD	
Baseline	94.27 ± 8.33	91.44 ± 10.70	0.294
10 minutes after the bolus	76.79 ± 4.39	81.02 ± 6.35	0.008
During laryngoscopy and intubation	104.0 ± 4.58	100.46 ± 10.05	0.109
Pinning of the skull	87.11 ± 6.56	86.83 ± 8.17	0.892
Skin Incision	81.17 ± 5.16	79.78 ± 7.71	0.446
Bone flap removal	77.28 ± 3.77	77.39 ± 6.66	0.939
Dura opening	75.06 ± 3.49	75.46 ± 7.96	0.817
One hour after dura opening	78.81 ± 7.93	75.11 ± 8.93	0.121
Two hours after dura opening	78.27 ± 5.55	75.56 ± 9.96	0.263
Dura closure	79.11 ± 4.94	75.42 ± 12.62	0.171
At extubation	102.07 ± 5.78	103.07 ± 9.82	0.719
Five minutes after extubation	88.92 ± 4.09	93.15 ± 6.55	0.007
30 minutes after extubation	82.37 ± 3.65	88.71 ± 7.27	<0.001
One hour after extubation	81.71 ± 4.35	85.37 ± 6.70	0.023
Two hours after extubation	83.96 ± 4.49	84.25 ± 7.58	0.865

However, MAP was within 20% of the baseline. MAP was comparable at other time points.

A significant decrease in emergence time was found in group D (9.19 ± 3.26) compared to group F (22.62 ± 15.85) (*P *< 0.001). NRS was found to be significantly lower in group D at eight hours (*P *= 0.005) and 12 hours (*P *< 0.001) post-extubation, whereas comparable at another time point.

Other parameters like modified Aldrete score, patient satisfaction, surgeon satisfaction, and length of hospital stay were comparable among the two groups, as shown in Table 4.

**Table 4:** Modified Aldrete score, patient satisfaction, surgeon satisfaction, and length of hospital stay comparison between two groups. *P *< 0.05 is considered significant. SD, standard deviation; *n*, number

Variables	Group D (*n *= 26)	Group F (*n *= 26)	*P*-value
	Mean ± SD	Mean ± SD	
Modified Aldrete score	9.61 ± 0.50	9.65 ± 0.49	0.779
Patient satisfaction score	5.0	5.0	-
Surgeon satisfaction score	2.85 ± 0.37	2.92 ± 0.27	0.395
Length of hospital stay (days)	5.0 ± 0.80	4.96 ± 0.77	0.861

No additional fentanyl bolus was required intraoperatively in either group. The time to the first request for analgesia in the postoperative period was 55.83 ± 22.84 minutes in group D and 58.95 ± 18.63 minutes in group F, demonstrating comparability (*P* = 0.547). We did not encounter any complications such as hypotension, hypertension, bradycardia, or tachycardia in the two groups.

## Discussion

This study was designed to compare dexmedetomidine versus fentanyl-based general anesthesia in patients undergoing elective glioma surgery. Our research's primary outcome was comparing perioperative hemodynamics between the two groups. The secondary outcome was to estimate and compare the doses of additional intraoperative fentanyl bolus, emergence time, modified Aldrete score, NRS, patient satisfaction, and surgeon satisfaction between the groups.

We observed that dexmedetomidine infusion started before surgery maintains hemodynamic stability throughout the study period and reduces the emergence time with comparable modified Aldrete score, patient satisfaction, and surgeon satisfaction compared to the fentanyl without any significant intraoperative complications.

Maintaining intraoperative hemodynamic stability during neurosurgical patients is essential because hypertension may lead to hemorrhage and vasogenic edema, which finally leads to an increase in intracranial pressure (ICP), and low blood pressure may result in cerebral ischemia in the areas of impaired autoregulation. Also, early emergence will help in detecting postoperative complications at the earliest. Hence, the focus should be on hemodynamic stability and early emergence during neurosurgery.

Earlier studies have shown that dexmedetomidine provides a blunting response during laryngoscopy, intubation, skull pinning, and extubation in various surgeries under general anesthesia, which was similar to the result of this study [1,10,12-14].

One meta-analysis showed evidence that dexmedetomidine as an anesthetic adjuvant during intracranial procedures leads to better perioperative hemodynamic control and less intraoperative opioid consumption, which is similar to the result of our study [2].

We observed comparable postoperative recovery profile scores (modified Aldrete scores) in both study groups, which is in alignment with one study in which the authors compared dexmedetomidine with remifentanil in adults having elective brain tumor excisions under balanced general anesthesia with endotracheal intubation [15].

Contrary to our study result, one of the studies in which dexmedetomidine was compared to sufentanyl during burr-hole surgery for chronic subdural hematoma found that dexmedetomidine shortened postoperative recovery time and had better patient and surgeon satisfaction [16]. Our study showed comparable patient and surgeon satisfaction scores in both groups, and there was no difference in the length of hospital stay in either group. The comparison of dexmedetomidine with sufentanyl in place of fentanyl with different doses might be the reason for varying results from our study.

In this study, no patients required additional fentanyl boluses in either group. However, a study compared dexmedetomidine versus fentanyl-based anesthetic protocols in patients undergoing elective neurosurgical procedures, and the researchers found that additional fentanyl boluses were required in the fentanyl group. This can be explained as they gave 0.5 mcg/kg/hour of fentanyl infusion, whereas in our study, we administered 1 mcg/kg/hour of fentanyl infusion [17]. In another study, the analgesic potency of fentanyl and dexmedetomidine was found to be comparable [18].

There are certain limitations to our study. We did not measure the plasma concentration of dexmedetomidine and fentanyl perioperatively. Patients with altered drug metabolism would have had lower or higher effective doses. We relied on hemodynamics to evaluate analgesia, but ideally, an analgesia nociception index monitor might have quantified the analgesic potential of dexmedetomidine and fentanyl. The cost-effectiveness of the drugs used is not studied in the present study. Context-sensitive half-lives did not guide the discontinuation of study drugs. Other limitations of this study include a single-center experience, a small sample size, and a nonrandomized study design.

## Conclusions

This study aimed to compare opioid-based (fentanyl) versus opioid-sparing (dexmedetomidine) general anesthesia for perioperative hemodynamic stability and postoperative recovery characteristics in patients undergoing glioma surgeries. This study concludes that dexmedetomidine can be used as an alternative to fentanyl in terms of perioperative hemodynamic stability, perioperative analgesia, patient satisfaction, surgeon satisfaction, and smooth early recovery from anesthesia. Randomized controlled trials with large sample sizes are required to verify the advantages of dexmedetomidine over fentanyl in glioma surgeries.
